# Salt Bridge Formation between the I-BAR Domain and Lipids Increases Lipid Density and Membrane Curvature

**DOI:** 10.1038/s41598-017-06334-5

**Published:** 2017-07-28

**Authors:** Kazuhiro Takemura, Kyoko Hanawa-Suetsugu, Shiro Suetsugu, Akio Kitao

**Affiliations:** 1 Institute of Molecular and Cellular Biosciences, The University of Tokyo, 1-1-1 Yayoi, Bunkyo, Tokyo, 113-0032 Japan; 2 Graduate School of Biological Sciences, Nara Institute of Science and Technology, Ikoma, Nara, 630-0192 Japan

## Abstract

The BAR domain superfamily proteins sense or induce curvature in membranes. The inverse-BAR domain (I-BAR) is a BAR domain that forms a straight “zeppelin-shaped” dimer. The mechanisms by which IRSp53 I-BAR binds to and deforms a lipid membrane are investigated here by all-atom molecular dynamics simulation (MD), binding energy analysis, and the effects of mutation experiments on filopodia on HeLa cells. I-BAR adopts a curved structure when crystallized, but adopts a flatter shape in MD. The binding of I-BAR to membrane was stabilized by ~30 salt bridges, consistent with experiments showing that point mutations of the interface residues have little effect on the binding affinity whereas multiple mutations have considerable effect. Salt bridge formation increases the local density of lipids and deforms the membrane into a concave shape. In addition, the point mutations that break key intra-molecular salt bridges within I-BAR reduce the binding affinity; this was confirmed by expressing these mutants in HeLa cells and observing their effects. The results indicate that the stiffness of I-BAR is important for membrane deformation, although I-BAR does not act as a completely rigid template.

## Introduction

Plasma membrane invaginations and protrusions are essential processes for endocytosis, cell migration, and other cellular dynamics. Bin Amphiphysin Rvs167 (BAR) domain superfamily proteins are often involved in such deformations of cellular membrane^1^. “Canonical” BAR domains^2^, hereafter called ‘BAR domain’ form a banana-shaped homodimer and interact with the membrane through their concave surfaces via charged amino acids. Extended Fes-CIP4 domain (F-BAR)^3–6^ adopts a more elongated structure that induces the formation of thicker membrane tubules. BAR and F-BAR are believed to deform membranes by molding the membrane structure to the BAR domain surface. Insulin receptor tyrosine kinase substrate of 53 kDa (IRSp53)^7–11^ is an adaptor protein comprising an N-terminal membrane binding domain, a CRIB motif that can bind to Cdc42^12, 13^, and a Src homology 3 domain bound to several actin regulators^8, 14, 15^. This membrane-binding domain of IRSp53 is classified as an Inverse-BAR domain (I-BAR), and found in five proteins in mammals, which are IRSp53, IRTKS, pinkbar, MIM, and ABBA^8, 16^. In contrast to other BAR domains, I-BAR forms a straight or “zeppelin-shaped” dimer and induces outward protrusions of the plasma membrane. Consistently, IRSp53 plays key roles in the formation of filopodia and lamellipodia^10^, which are related to several essential biological processes such as neurite extension^12^, dendric spine formation^17–20^, myogenic differentiation^21^, lens formation^22^, and tumor invasion^23, 24^.

The molecular mechanisms by which the BAR domain causes membrane deformation have been extensively studied computationally by molecular dynamics simulation (MD) of the BAR domain with N-terminal amphipathic helices (N-BAR) of amphiphysin^25–32^ and endophilin^33–36^. In N-BAR, insertion of the helices into one side of the membrane is expected to curve the membrane into a convex shape. A single N-BAR domain deforms the membrane locally and multiple N-BAR domains are required to induce global membrane deformation, i.e., tubulation and vesiculation; Multi scale simulation results showed that membrane curvature depends on the concentration and arrangement of the N-BAR domains^27, 29, 31^. Mesoscopic simulations showed that N-BAR induces tubulation at low concentration and causes vesiculation at high density^26, 30^. Especially, the arrangement of endophilin N-BAR was investigated using cryo-electronmicroscopy and coarse-grained MD simulation^35, 36^. The increase in membrane curvature caused by a single N-BAR induces the binding of other N-BAR domains, which may further induce high-curvature in the membrane^35^. Coarse-grained MD simulations also showed that N-BAR tends to assemble linearly when the surface tension is low, whereas high surface tension inhibits interactions between N-BAR domains and alters the geometry of N-BAR association^37^. Electron paramagnetic resonance with site-directed spin labelling studies suggested that vesicle binding of the N-BAR protein amphiphysin is mediated by shallow insertion of amphiphathic N-terminal helices whereas the interaction with tubes involves deeply inserted N-terminal helices with concave surface of the BAR domain^38^. All-atom MD indicated that F-BAR changes the curvature of the membrane via scaffolding mechanism even though F-BAR itself is flexible^39^. These results suggest that distinct BAR domains may use different binding and membrane bending mechanisms.

The molecular mechanisms of I-BAR tubulation were investigated using point and multiple mutations of Lys and Arg residues for IRSp53^7^. Several Lys residues in each protomer are believed to form a “lipid binding line”, generating two binding lines in the domain. Only one lipid binding line likely binds to the membranes, given that the simultaneous binding of two lines may be structurally difficult if I-BAR is completely rigid^7, 8, 40^. Although I-BAR domains in different proteins including IRSp53, pinkbar, and MIM are structurally similar^11, 41–43^, they induce tubules with diameters^9^ ranging from 40 to 60 nm, significantly smaller than the value ~95 nm estimated from the structure of I-BAR^40^. To explain the variations in the diameter, PI(4,5)P_2_ was suggested to induce protein-dependent clustering and insertion of hydrophobic amino-acid residues for the I-BAR domain from MIM^9^, however such mechanisms appeared to be absent for I-BAR from IRSp53 to induce membrane deformation^7^. With an assay in which I-BAR domains were encapsulated in giant unilamellar vesicles connected to membrane nanotubes, it was shown that I-BAR senses negative curvature and is enriched at a curvature, 0.055 nm^−1^(=36 nm diameter), and has significant mechanical effects on curved membranes^44^. All-atom and coarse-grained molecular dynamics simulations suggested that I-BAR interacts and bends negatively charged phospholipids^45^.

The molecular mechanisms by which I-BAR binds to and deforms a lipid membrane was investigated here using all-atom MD simulation, binding energy calculations and mutational effects on formation of filopodia on HeLa cells. Multiple MD simulations starting from distinct membrane-unbound states showed spontaneous binding of an I-BAR domain dimer to the membrane and the induction of increased membrane curvature. Analysis of the binding energy indicated that salt-bridge formation between I-BAR and the lipid headgroups provides the driving force to induce membrane deformation. Furthermore, the formation of salt bridges increases the local density of the lipids and deforms the membrane. In addition, the importance of intramolecular salt-bridges within I-BAR is shown by comparing the binding affinities of mutants and the effects of these mutations on filopodia observed in HeLa cells. Our findings indicate that I-BAR must exhibit some degree of stiffness in order to cause membrane deformation, although I-BAR is a quite flexible and does not act as a rigid template.

## Results

### Strong Directivity of I-BAR in the Binding to Membrane

**Figure 1:**
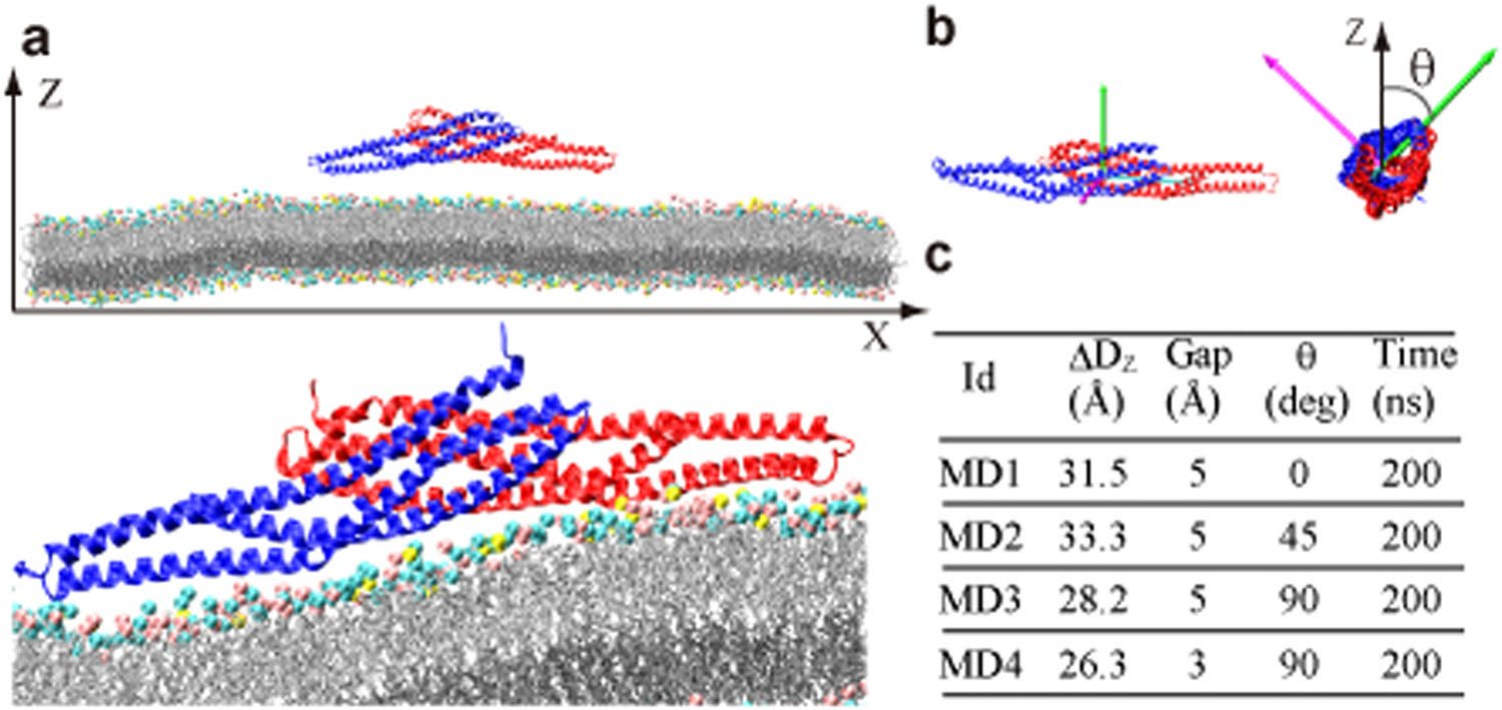
Simulated system containing I-BAR and a lipid bilayer membrane. (**a**) The initial arrangement of I-BAR (upper panel) and a snapshot after 200-ns MD (lower panel) in the MD1 simulation. Pink, cyan, and yellow spheres represent the headgroups of DOPC, DOPE, and DOPS, respectively. (**b**) Principal axes of inertia of I-BAR (three arrows). The angle θ is defined as the angle between the second principle axis of inertia (green) and the Z-axis; the latter is initially parallel to the membrane normal in MD1. (**c**) Initial conditions of the four MD simulations of I-BAR–membrane systems (MD1–4). *ΔD*_*Z*_ is defined in the main text. ‘Gap’ is the minimum heavy atom pair distance between I-BAR and the membrane along the Z-axis. The molecular graphics was created by VMD^57^.

The process by which I-BAR binds to a lipid bilayer membrane was observed using four MD simulations (Referred as MD1–4. Supplementary Movies 1–4). Each simulation began with a distinct I-BAR initial positions, with an *θ* and inter-molecular center of mass distance *D*_*Z*_ along the membrane normal (Z-axis) (Fig. 1). *D*_*Z*_ represents the actual distance and *ΔD*_*Z*_ indicates the relative value from the completely bound state, which was defined as the global free energy minimum obtained by free energy calculation in MD1 (see below); those in MD2, MD3, and MD4 were defined as average *D*_*Z*_ values from the last 10-ns of 200-ns MD simulations. The conditions used in I-BAR binding experiments to liposome^7^ were replicated by constructing the simulated lipid bilayer membrane from dioleoyl-phosphatidylcholine DOPC and dioleoyl-phosphatidylethanolamine (DOPE) and dioleoyl-phosphatidylserine (DOPS) at a ratio of 4:4:1. DOPC and DOPE are neutral phospholipids whereas DOPS is negatively charged. MD simulation of solutions containing I-BAR alone and membrane alone were also conducted. This lipid bilayer model does not include multivalent phosphoinositides. We used the lipid bilayer with DOPC/DOPE with monovalent negatively charged lipid (DOPS) as a minimal model, because I-BAR from IRSp53 was reported to function in the absence of the phosphoinositide and addition of phosphatidylinositol triphosphate (PIP_3_) did not further increased the I-BAR binding^7^. In the MD, there appeared to be no specific binding site of PS on the surface of the I-BAR domain, and therefore, it is suggested that phosphoinositide would also bind to the I-BAR domain mainly through electrostatic interaction without specific binding pockets. However, in the case of the I-BAR domain from MIM, PI(4,5)P_2_ was suggested to play an important role in membrane deformation^9^, and therefore the effects of phosphoinositides on the I-BAR domains of IRSp53 and MIM will be examined in the future.

**Figure 2:**
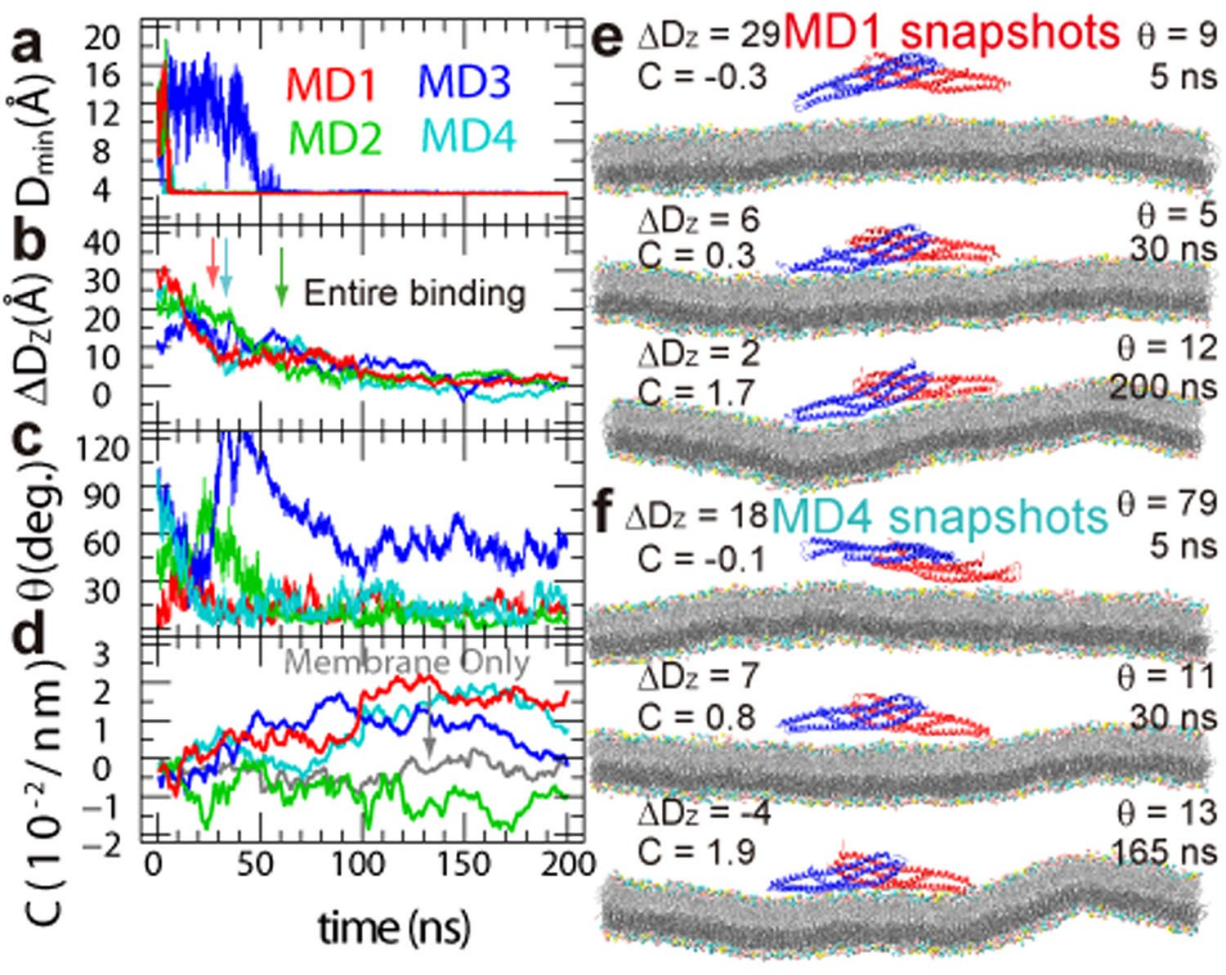
Time evolution of I-BAR binding to the lipid bilayer membrane. (**a**) Minimum heavy atom pair distance between the I-BAR domain and the membrane, (**b**) I-BAR–membrane distance *ΔD*_*Z*_, (**c**) angle θ, and (**d**) curvature *C*_*ave*_ as a function of MD simulation time. (**e,f**) representative snapshots from (**e**) MD1 and (**f**) MD4. The molecular graphics was created by VMD^57^.

I-BAR spontaneously made first contact with the membrane at 4.8 (MD1, contacting residue: K152), 5.4 (MD2, D159 and Q163), 1.8 (MD3, R114) and 1.7 ns (MD4, R114) (Fig. 2a), but these first contacts were not always stable. Once more than ten residues were in contact with the lipids, the bindings became stable using residues at one end of the I-BAR homodimer (Table S1). K156 and K160 interacted with the lipids in all the simulations at this stage. As the simulation progressed, more residues gradually interacted with the lipids until the entire I-BAR stably bound to the membrane. After 27.3 (MD1), 60.2 (MD2), and 36.4 ns (MD4) (Fig. 2b), I-BAR bound in almost the same orientation (Fig. 2c) and through essentially the same interface. Regardless of its initial orientations, I-BAR reoriented relatively quickly (<100 ns) to the orientation consistently obtained by the end of the simulation, indicating that I-BAR is strongly directed to the membrane. Reorientations of I-BAR were also observed in coarse-grained MD simulations^45^. In this case, however, I-BAR bound to the membrane with the opposite orientation in which C-terminal residues directed toward the membrane as shown in “system 3”^45^. Since the C-terminal of the I-BAR is connected to other domains of IRSp53, this binding orientation may be unrealistic. The binding of both ends of I-BAR to the membrane drastically increased the curvature of the membrane (Fig. 2d). In this study, the curvature is defined as being positive if the membrane is bent towards I-BAR. The curvature averaged over the I-BAR binding region of the membrane (*C*_*ave*_) was in the range 1.2 ~ 2.3 × 10^−2^ nm^−1^ after 100 ns in MD1, which is considerably higher than the curvature observed in membrane-only simulation (See SI Text for the method for calculating *C*_*ave*_). Selected snapshots from MD1 and MD4 are shown in Fig. 2e,f. The I-BAR domain as a whole, and especially the tips around Gly150 (Supplementary Fig. S1), is very flexible in solution and in the unbound state, and is flatter than the structure observed crystallographyically^7^ (see later sections).

### Electrostatically-driven I-BAR Binding and Membrane Deformation

**Figure 3:**
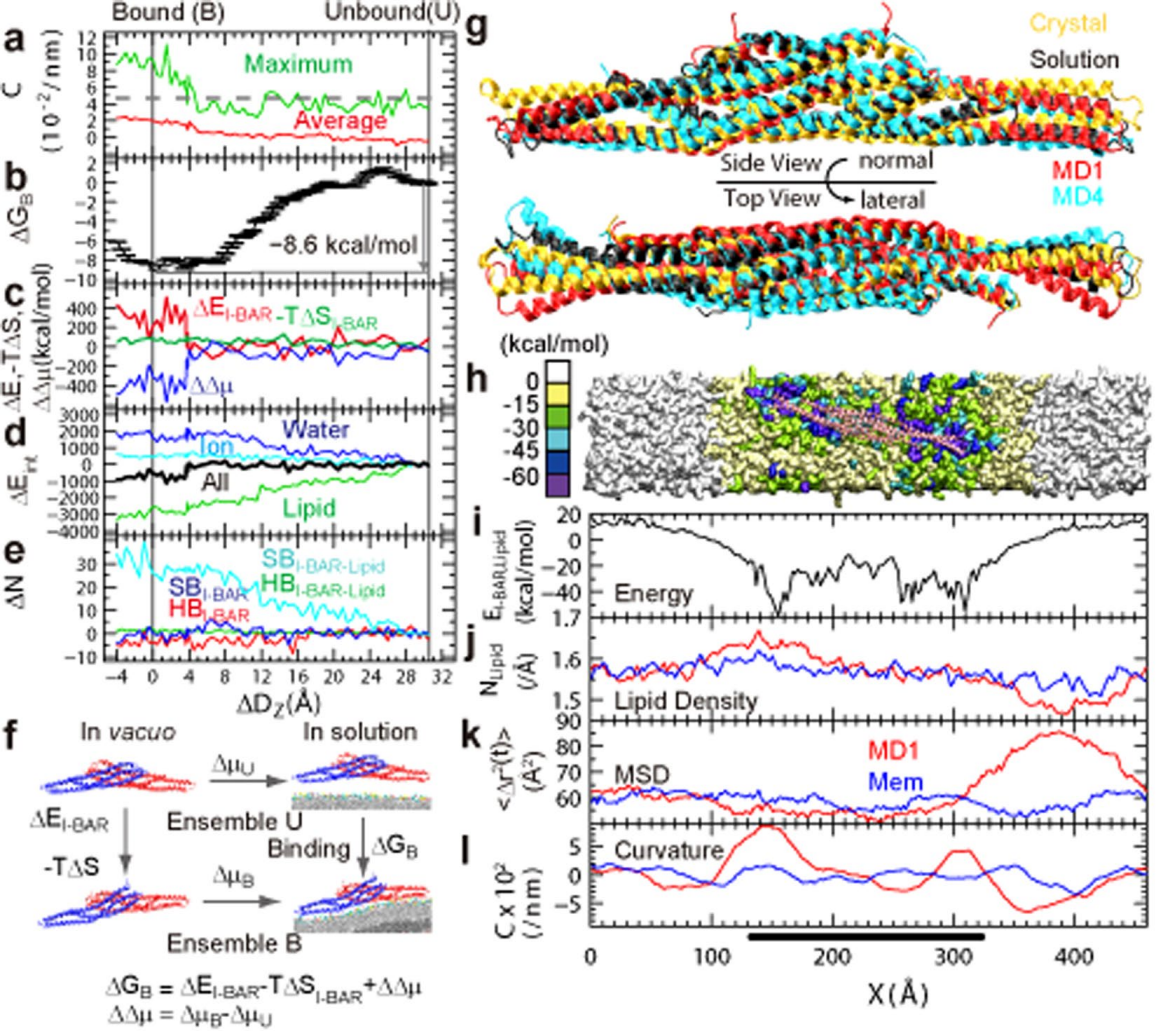
Free energy analysis of I-BAR binding. (**a**) Curvature, (**b**) binding free energy *ΔG*_*B*_, (**c**) free energy components *ΔE*_*I-BAR*_, −*TΔS*_*I-BAR*_, and *ΔΔμ*, (**d**) components of *ΔE*_*int*_, (**e**) the number of hydrogen bonds and salt bridges within I-BAR and between I-BAR and the lipids, as a function of *ΔD*_*Z*_. In (**a**), the broken line shows experimentally deduced curvature^9^. (**f**) Thermodynamic cycle of binding/unbinding. (**g**) The structures of I-BAR in the crystal, in solution, and from MD1 and MD4. (**h**) Interaction energy between I-BAR and each lipid headgroup mapped on the XY plane. (**i**) Interaction energy between I-BAR with the lipid headgroups integrated along the Y-axis. (**j**) Lipid local density (N_lipid_), (**k**) mean square displacement (MSD, <*Δr* ^*2*^ *(t)*>) of the lipids in bilayer lateral direction at time *t* = 10 ns, and (**l**) curvature (C) as a function of the X coordinate. The horizontal bar in the bottom indicates the position of I-BAR. The results shown in (**h**) to (**l**) were obtained from 107~137 ns in MD1 and 140~170 ns in membrane only system. Center of mass for the headgroup was used to calculate lipid density, MSD, and curvature. The lipid densities and MSDs were averaged over the range of ±48.3 Å. The molecular graphics was created by VMD^57^.

The maximum and average curvatures, *C*_*max*_ and *C*_*ave*_, and free energy, *ΔG*_*B*_, of I-BAR binding were calculated as a function of the center of mass distance *ΔD*_*Z*_ between I-BAR and the membrane (Fig. 3a,b). The origin of *ΔD*_*Z*_ was set to the global free energy minimum, and the second minimum was positioned at *ΔD*_*Z*_ = 3.5 Å. *ΔG*_*B*_ is *−*8.6 kcal/mol at *ΔD*_*Z*_ = 0, compared to a value at *ΔD*_*Z*_ = 30.5 Å. The free energy differences are relatively small (~2 kcal/mol) in the range *ΔD*_*Z*_ = *−*3~8 Å, and the entire I-BAR is bound to the lipid bilayer. A decrease in *ΔG*_*B*_ correlated with an increase in curvature (Fig. 3a,b). The macroscopic curvature of the membrane deduced from the diameter of the membrane tubule induced by I-BAR observed using transmission electron microscopy^9^, *C*_*exp*_, is 4.7 × 10^−2^ nm^−1^ (broken line in Fig. 3a). The curvatures, *C*_*max*_ = 9.2 × 10^−2^ nm^−1^ and *C*_*ave*_ = 2.0 × 10^−2^ nm^−1^ at the binding free energy minimum correspond to 22 and 100 nm in diameter, respectively. *C*_*max*_ roughly indicates the maximum limit of microscopic deformation that a single I-BAR can induce locally, and *C*_*ave*_ gives a slightly more macroscopic average. The membrane deformation observed in this work can be considered as an initial process of the global deformation induced by the binding of the first I-BAR. Furthermore, given that *C*_*exp*_ provides a macroscopic value due to the binding of multiple I-BAR, the order *C*_*max*_ > *C*_*exp*_ > *C*_*ave*_ is reasonable.

The mechanism underlying binding and deformation were investigated by conducting free energy component analysis, *ΔG*_*B*_ = *ΔE*_*I-BAR*_ −  *TΔS*_*I-BAR*_ + *ΔΔμ*, based on the thermodynamic cycle (Fig. 3c–f). Here, ensembles *U* and *B* indicate the I-BAR structural ensembles generated as membrane unbound (*ΔD*_*Z*_ = 30.5 Å) and bound states (0 Å) in solution, respectively. *Δμ*_*U*_ and *Δμ*_*B*_ (*ΔΔμ* = *Δμ*_*B*_  − *Δμ*_*U*_) represent the free energy of I-BAR solvation when I-BAR is transferred from vacuum to a mixed solvent consisting of water, ions and lipids, and I-BAR is in the unbound or bound states, keeping the I-BAR structure in ensembles *U* and *B* as in solution, respectively. *ΔE*_*I-BAR*_ and *ΔS*_*I-BAR*_ are defined as the difference of the average conformational energies and entropies of I-BAR *in vacuo* between ensembles *U* and *B*, respectively. Similar to *ΔG*_*B*_, these energy terms were obtained as a function of *ΔD*_*Z*_ (Fig. 3c). A drastic change in membrane curvature occurred simultaneously with a change in energy component at around *ΔD*_*Z*_ = 4 Å after the entire I-BAR bound to the membrane. The membrane curvature increased as the global minimum was approached. The energy components changed at *ΔD*_*Z*_ = 0 Å: *ΔΔμ* = *−*316.3, *ΔE*_*I-BAR*_ = 226.0 and *−TΔS*_*I-BAR*_ = 81.7 kcal/mol. The increase in *−TΔS*_*I-BAR*_ during the membrane binding process is consistent with I-BAR being very flexible in the unbound state but becoming less flexible as it bounds tighter to the membrane.

To examine *ΔΔμ*, we calculated the I-BAR interaction energies *ΔE*_*int*_ with the water molecules, ions, and lipids (Fig. 3d). This approach was justified because the total *ΔE*_*int*_ highly correlated with *ΔΔμ* ^46^ as seen in Fig. 3c,d. The binding of I-BAR to the lipid bilayer resulted in an increase in its *ΔE*_*int*_ with the waters and ions, consistent with dehydration and counterion dissociation from the I-BAR surface. However, although counterion dissociation accompanies the increase in *ΔE*_*I-BAR*_, there was a significantly larger energy decrease due to interactions between I-BAR and the lipids (Fig. 3d). This energy decrease was mainly caused by the formation of ~ 30 salt bridges between I-BAR and the lipid headgroups (Fig. 3e), consistent with previous results showing that mutations in the positively charged residues of I-BAR decrease binding affinity^7^. Here, a salt bridge was considered to be formed when the distance between any of the nitrogen atoms of basic group and the oxygen atoms of acidic group was within 3.2 Å.

### I-BAR is Relatively Flat and Flexible

The I-BAR crystal structure is a somewhat curved, whereas the MD calculations showed that I-BAR adopts a flatter structure in solution (Fig. 3g). This flat structure makes it difficult to understand how I-BAR can cause membrane curvature if I-BAR acts as a rigid template. The tight binding of I-BAR with the membrane restricts the fluctuations of I-BAR compared to when I-BAR is in solution (Supplementary Fig. S1). After binding to the membrane, the structural variations of I-BAR along the membrane bilayer normal direction were relatively restricted, but large variations and fluctuations were observed along the lateral direction (Fig. 3g and Supplementary Fig. S2). Although the I-BAR structures in MD1 and MD4 are significantly different in the lateral direction (Fig. 3g), both caused the membrane to curve considerably (Fig. 2d). I-BAR remains flexible even after tight binding to a lipid membrane, especially along the lateral direction (Supplementary Fig. S2). Therefore, I-BAR does not act as a completely rigid template, and so shape is not the deciding factor inducing membrane deformation. Principal component analysis also showed that I-BAR in solution is intrinsically more flexible along the lateral direction and stiffer along the normal direction (Supplementary Fig. S3). Although I-BAR is relatively flexible, this anisotropy may be related to the stiffness of I-BAR and be important for inducing membrane deformation along the membrane normal.

### Salt Bridge Formation Induces an Increase in Local Lipid Density and Membrane Curvature

The lipid headgoups that strongly interacted with I-BAR were situated around the tips of the dimer as shown by the interaction energy between each lipid headgroup and I-BAR (Fig. 3h,i). DOPS binds more tightly to the I-BAR than DOPC and DOPE as shown in Supplementary Fig. S4. After tight binding of I-BAR to the membrane in MD1, the local density of the lipids was elevated around the I-BAR-bound region (Fig. 3j) and the lateral mean-square displacement (MSD) of the lipids was reduced compared to the membrane without I-BAR (Fig. 3k). The local curvature reached a maximum value of 8.6 × 10^−2^ nm^−1^ at X = 142 nm (Fig. 3l), which concurs with the lowest energy minimum as well as the highest lipid density peak. Therefore, it is concluded that the salt bridge formation between I-BAR and the lipids increased the local density of the lipids, which in turn induced deformation of the membrane. The lowest minimum of negative curvature at X = 361 nm is considered to be an artifact of the periodic boundary condition, which compensates the positive membrane curvature induced by I-BAR. The local density of the lipids around the negative peak was significantly lower than the density of other regions, and MSD is considerably increased.

### Contributions of Charged Residues to Binding and Stability

**Figure 4:**
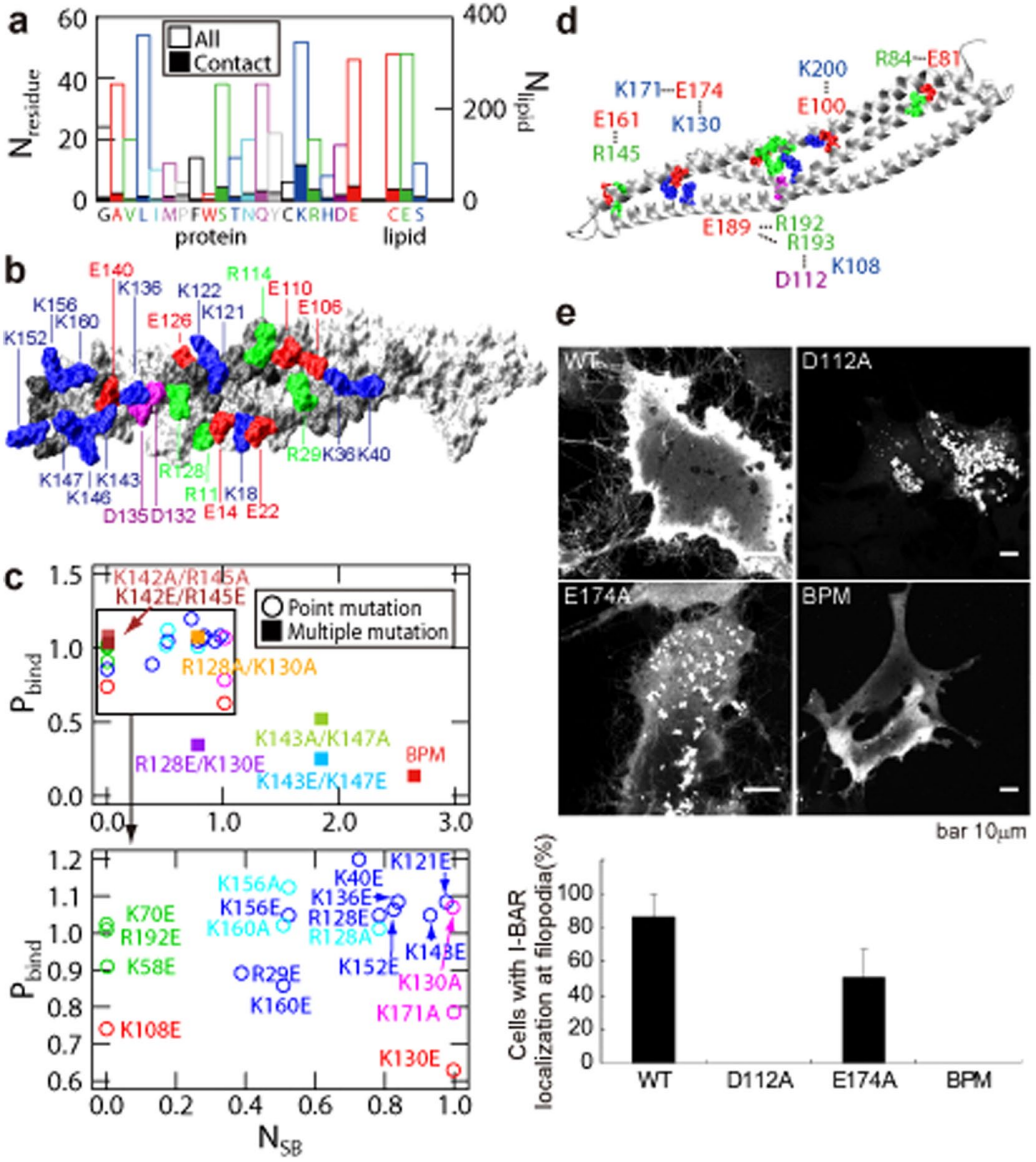
Effects of mutations on the membrane. (**a**) The number of each amino acid type and the number of each lipid type involved in binding. Open boxes indicate the total number of amino acid residues and lipids in the upper layer of the bilayer. Filled boxes represent the number of residues and lipids making contact at the I-BAR/membrane interface. (**b**) Positively and negatively charged residues located on the membrane binding surface. (**c**) Binding affinities relative to the wild-type as a function of the number of salt bridges that the wild-type can make with the lipid headgroups. The number of intra-molecular salt bridges for K108, K130, and K171 which do not make salt bridge with the lipid headgroup are also shown. (**d**) Intra-molecular salt bridges essential for I-BAR stability. (**e**) Effects of D112A, E174A and BPM mutations compared to the wild-type on HeLa cells observed by fluorescence microscopy with GFP fusion I-BAR and percentage of cells with I-BAR localization at filopodia. BPM: K142A/K143A/R145A/K146A/K147A. The molecular graphics was created by VMD^57^.

I-BAR is a basic-charged protein domain containing many charged amino acid residues (+*8e* in total): 52 Lys and 20 Arg, and 18 Asp and 46 Glu in the 232 × 2 residues per dimer (Fig. 4a,b). When an amino acid is considered to contact the lipid if the distance between any heavy-atom pair is 5 Å or less, the residue contacting the lipids most frequently is Lys, which form 28.5 ± 2.4 contacts with lipids and 15.4 ± 2.9 salt bridges at around the global free energy minimum (the values after ± shows the standard deviation). Glu residues made the second largest number of contacts (13.2 ± 1.9 contacts, 4.2 ± 1.2 salt bridges), followed by Ser, Arg (8.1 ± 2.0 salt bridges), Gln, and Asp (1.1 ± 0.6 salt bridges). A total of 28.7 ± 3.8 salt bridges were formed between bound I-BAR and the lipids. DOPC, DOPE, and DOPS were in contact with 23.5 ± 1.4 (8.2 ± 1.5 salt bridges), 22.8 ± 3.1 (14.1 ± 3.2) and 7.2 ± 0.4 (6.5 ± 1.8) amino acid residues, respectively. The PC and PE headgroups are overall neutral whereas that of PS is negatively charged. Since the simulated membrane comprises four times as much DOPC and DOPE as DOPS, DOPS appears to interact more with I-BAR than do the other lipids. These observations agree with the experimental results that I-BAR forms more interactions with DOPS rather than with other lipids (DOPC and DOPE) and that the mutations of positively charged residues reduces the binding affinity of I-BAR to lipid membranes^7^.

The binding affinity of point and multiple mutations of Lys and Arg residues relative to the wild-type was previously measured by ELISA experiments^7^, and the correlation of binding affinity with the number of salt bridges between the mutated residues and lipids in wild-type was examined (Fig. 4c). Basic patch mutant (BPM) designates the K142A/K143A/R145A/K146A/K147A mutant. The binding affinities of the multiple mutations negatively correlated with the number of salt bridges, but no such correlation was seen with the point mutations. K40, K121, R128, K136, K143, and K152 formed salt bridges with the lipids but their mutation to Glu did not affect binding affinity substantially (blue data points in Fig. 4c). The effects of R128A are also negligible (not shown in Fig. 4c). Since ~30 salt bridges were formed in total (Fig. 3e), point mutations do not reduce the number of salt bridges drastically; this could explain why they have little effects on binding affinity.

K108, K130, and K171 (red points in Fig. 4b,c) did not form salt bridges with lipids, yet the K108E, K130E and K171A mutations considerably affect the binding affinity. These residues form intra-molecular salt bridges in the wild-type (Fig. 4d), and thus their mutations should affect the stability of I-BAR rather than its interactions with lipids. Interestingly, K130A has little effect: even though the K130-K174 salt bridge is broken, K171-E174 salt bridge should be maintained. The K130E mutant might break these intra-helical salt bridges by forming an inter-helical E130-K171 salt bridge. This hypothesis is supported by experiments using D112A and E174A mutants (Fig. 4e), which showed reduced localization at filopodia compared to the wild-type I-BAR. Note that Fig. 4e does not represent the percentage of cell with filopodia but that of cells with I-BAR localization at filopodia. Because D112A and E174A mutants were not localized in filopodia, these mutants are considered to be unable to contribute to filopodia formations. Dotted signals in D112A and E174A mutants can be interpreted that the expressed proteins remained insoluble or were improperly fold, and then aggregated. The more notable effect of D112A mutant compared to E174A mutant may be related to the location of the mutated residue: the elimination of the salt bridges closer to the protein core could affect I-BAR stability more than the mutation of residue 174, which situated near the tip of I-BAR. These mutations would be expected to destabilize I-BAR by eliminating the inter-helical salt bridges formed by D112-R193 and K130-E174-K171 interactions (Fig. 4d).

## Discussion

In this study, we observed the spontaneous binding of I-BAR to a membrane using MD simulations. Regardless of the initial orientation I-BAR bound to the membrane through the same interface. This strong directivity, evident within 100 ns, is due to strong electrostatic interactions between I-BAR and lipid headgroups. We had anticipated that only one tip of the I-BAR dimer binds to the membrane, but found that both ends of the I-BAR bound to the membrane sequentially after first contact at the one tip of the dimer in this work. The entire binding of I-BAR to the membrane were observed in MD1, MD2, and MD4 within 200 ns. Although the orientation of I-BAR and the number of interactions between I-BAR and the membrane in MD2 were not significantly different from those in MD1 and MD4, the observed curvature in MD2 at 200 ns was not yet positive. One possible reason is that the entire binding in MD2 happened ~30 ns later compared to those in MD1 and MD4, as indicated by the arrows in Fig. 2b. Another possible reason is that, when the entire I-BAR bound to membrane, the membrane curvature in MD2 was negative, while those in MD1 and MD4 were happen to be positive (Fig. 2d, Supplementary Movie S1–4). Considering above, we expect that longer time is required to convert the negative membrane curvature to positive in MD2, compared to the time spent in MD1 and MD4.

Global membrane deformations are induced by multiple BAR domains^16^. I-BAR domains were also reported^44^ to be enriched at a curvature ~ 0.055 nm^−1^, although the arrangements of them are still unclear. Thus, the membrane deformation observed in this work should be considered as an initial process of the global deformation induced by the binding of the first I-BAR. More complicated factors such as arrangements and densities of I-BAR and effects of phosphoinositide should be considered for complete understanding of the mechanism underlining the global deformation. We speculate that interactions among multiple I-BAR domains may contribute to increase the binding of I-BAR to the membrane. Simulations including such conditions are currently under consideration.

The MD results showed that I-BAR is relatively flexible both in solution and on the membrane, adopting a flatter shape compared to the crystal structure (Fig. 3g). Therefore, I-BAR can induce the curvature change larger than expected given its flat structure. The formation of salt bridges between I-BAR and lipid decreased the interaction energy between them (Fig. 3h,i) and increased the local density of the lipids (Fig. 3j), resulting in significantly deformation of the membrane into a concave shape (Fig. 3l). The membrane curvature peaks are situated at the tips of I-BAR (Fig. 3h,i,l), which also indicates that the I-BAR shape is not the deciding factor inducing membrane deformation. This indicates that electrostatic interactions are essential both for I-BAR to deform the membrane and for its directive binding with the membrane. The salt bridge analysis by MD and mutational experiments showed that the salt bridges between I-BAR and the lipids, as well as within I-BAR, are essential for membrane deformation. Since ~ 30 salt bridges are formed between I-BAR and the lipid headgroups, it is reasonable that single mutations of I-BAR surface residues generally have no notable effects on membrane binding affinity unless inter-helical salt bridges are eliminated, in which case the effect is severe. Likewise, multiple mutations considerably affect the binding affinity (Fig. 4b,c). These findings indicate that I-BAR must be somewhat stiff in order to deform the membrane although I-BAR is overall flexible and does not act as a rigid template. This is consistent with our observation that I-BAR is more flexible along the lateral direction and stiffer along the normal direction.

Conformational changes of an F-BAR domain have been carefully investigated using MD^39^. The structures of the membrane-bound F-BAR are considerably different from the structures in the absence of lipid along both the normal (α angle in the reference) and lateral (θ) directions, whereas there is no notable difference between different membrane-bound F-BAR domains and among F-BARs in the absence of lipids. In contrast, the I-BAR structures in MD1 and MD4 are very different but caused a similar extent of membrane curving, suggesting that I-BAR is more flexible than F-BAR. In the case of N-BAR, Lyman *et al*. conducted MD simulations of N-BAR and lipids and reported that a considerable amount of water remains between the membrane and the positively charged concave face of N-BAR, even when it is tightly bound to the membrane^32^. We also observed that some water molecules remained at the binding interface during the binding and curving of the membrane. Not all of the BAR domain superfamily proteins appeared to function as a rigid template for membrane curvature as first expected from the structural diversity between BARs and F-BARs^47^. It is possible that the BAR domains have some bending capacity, which should be affected by the bending and stiffness of the membrane. The flexibility of the BAR domains might be important for generating relatively large subcellular structures such as filopodia and lamellipodia, both of which IRSp53 can localize^7^.

## Methods

### Molecular Dynamics Simulation

A lipid bilayer membrane consisting of 640 DOPC, 640 DPOE and 160 DOPS was constructed to mimic the liposome used in the experiments (SI Text)^7^. The X-ray structure of the I-BAR domain dimer (1WDZ.pdb)^7^ was placed above the equilibrated membrane in the arrangement shown in Fig. 1. The system containing membrane only was simulated for 200 ns. The I-BAR–membrane and membrane-only systems contain c.a. 720,000 atoms, including TIP3P(CHARMM) water and 0.1 M KCl in a box 470 × 90 × 150 Å^3^. A simulation of I-BAR in solution was conducted in a box for of 190 × 70 × 100 Å^3^ for 200 ns. MD simulations were performed using NAMD 2.9^48^. The CHARMM 22^49^ and 27^50^ force fields were used for the protein and lipids, respectively. The systems were brought to thermodynamic equilibrium at 300 K and 1 atm, using a Langevin thermostat and barostat under semi isotropic pressure control for the membrane containing systems and isotropic pressure control for I-BAR in solution. Equations of motion were integrated using a time step of 2 fs. The long-range Coulomb energy was evaluated using the particle mesh Ewald method.

### Free Energy Analysis

The free energy profile of the I-BAR binding *ΔG*_*B*_ was calculated using umbrella sampling conducted with NAMD 2.9^48^ and by the multiple Benette acceptance ratio method with pymbar^51^. *ΔD*_*Z*_ was defined as a reactive coordinate of the umbrella sampling, and a harmonic umbrella potential with a force constant of 50 kcal/mol/Å^2^ was imposed. MD1 snapshots whose *ΔD*_*Z*_ values were closest to the specified values (−4.0 to 30.5 Å every 0.5 Å, 3.6 to 3.9 Å every 0.1 Å) were selected as the initial structures for umbrella sampling. A 1-ns equilibration MD run and a 10-ns umbrella sampling simulations was conducted for each snapshot. We confirmed the convergence of the umbrella sampling simulation by monitoring obtained free energies from the independent simulations for 2, 4, 6, 8 and 10 ns (Supplementary Fig. S5). Results obtained from equal or longer than 6-ns umbrella sampling simulations were not much different and the results obtained from 10-ns simulations were used for analysis. *S*_*I-BAR*_ was calculated as the sum of the translational, rotational and internal motion entropies of I-BAR (see Supplementary Text)^52–55^. Effective frequencies of principal modes^56^ were used to calculate the internal motion entropy.

### Observation of Filopodia in HeLa Cells by Fluorescent Microscopy

The I-BAR domain of IRSp53 was expressed with green fluorescent protein as described in the ref. 7. HeLa cells were cultured in Dulbecco’s modified eagle medium supplemented with 10% fetal calf serum. Transfection was performed with the Lipofectamine LTX and PLUS reagents (Invitrogen) according to the manufacturer’s protocols. After 24 h of transfection, the cells were fixed and then observed under a confocal microscope (Olympus FV1000D) under a 100x oil immersion objective NA = 1.45 (Olympus)

## Electronic supplementary material

Movie S1

Movie S2

Movie S3

Movie S4

Supplementary Information
