# Supplementary material for: Salt Bridge Formation between the I-BAR Domain and Lipids Increases Lipid Density and Membrane Curvature
Source: Sci Rep. 2017 Jul 28;7:6808. doi: 10.1038/s41598-017-06334-5 (PMC5533756; doi:10.1038/s41598-017-06334-5)
Supplement: Supplementary file 5 — Supplementary Information [file 41598_2017_6334_MOESM5_ESM.pdf]

Salt Bridge Formation between the I-BAR Domain and Lipids Increases Lipid Density and Membrane Curvature

Kazuhiro Takemura[a], Kyoko Hanama-Suetsugu[b], Shiro Suetsugu[b], and Akio Kitao[a*]

[a]Institute of Molecular and Cellular Biosciences, The University of Tokyo, 1-1-1 Yayoi, Bunkyo, Tokyo 113-0032, Japan

[b]Graduate School of Biological Sciences, Nara Institute of Science and Technology, Ikoma, Nara 630-0192, Japan

Preparation of lipid bilayer membrane for MD.

A lipid bilayer membrane was first generated as a system containing 720×2-layers DOPC molecules in solution and then equilibrated. To mimic the experimental conditions [1], 320 and 80 DOPCs in both of upper and lower leaflet were randomly picked up and replaced by DOPE and DOPS, respectively. The obtained lipid bilayer system was farther equilibrated for 10-ns before arranging the I-BAR domain in the positions shown in Fig.1C.

Calculation of membrane curvature.

The center of mass position of the lipid head groups in upper leaflet were employed to calculate the membrane curvature. To calculate the curvature at a certain point X = a, X-Z profiles were fitted to cubic function in the range X = a ± 48.3 Å. Here the curvature is defined as being positive if the membrane is bent towards I-BAR. C_{ave} (Fig. 2A) was obtained as the average curvatures over the I-BAR domain binding range. C_{max} is defined as the maximum curvature within this range.

Calculation of I-BAR entropy.

Entropic contributions of I-BAR were calculated as a sum of translational, rotational and internal motion, which are respectively defined as[2-4]

$$S^{trans} = k_B \ln\left\{ \frac{\left(2\pi M k_B T\right)^{3/2}}{h^3} e^{5/2} V \right\}, \tag{1}$$

$$S^{rot} = k_B \ln\left\{ \frac{\left(2\pi k_B T\right)^{3/2} \left(I_X I_Y I_Z\right)^{1/2}}{h^3} e^{3/2} 8\pi^2 \right\}, \tag{2}$$

$$S^{int} = \sum_i k_B \left\{ \frac{1}{\alpha_i (\exp \alpha_i^{-1} - 1)} - \ln\left(1 - \exp(-\alpha_i^{-1})\right) \right\} \tag{3}$$

where $\alpha_i = 2\pi k_B T / h\omega_i$, k_B, h, T, M and V are Boltzmann constant, Planck constant, absolute temperature, mass of solute and volume (1 mol/l), respectively. I_X, I_Y and I_Z represent solute principal moments of inertia. Eq. (1) can be obtained for a system undergoing normal mode vibrations for each normal mode with angular frequency ω_i as described in the main text.

Table S1. List of Residues in Contact with the Lipids in the Initial State.

System	Residues
MD1	K121 K136 A139 E140 K143 K147 K152 P154 K156 K160 Q163 T164
MD2	K136 K143 L144 K147 S148 K152 P154 Q155 K156 K160
MD3	T115 A118 K122 E126 K130 K136 E140 K156 D159 K160 Q163 D166
MD4	Q107 E110 R114 T115 S148 Q155 K156 K160 Q163 K171 E174

Initial binding is defined when equal to or more than 10 residues make contact with the lipids. A residue is regarded as contacted with lipid when any of heavy atom is within 5 Å of lipids. Residues colored in red made first contact with lipid in the initial bindings. Residues colors in blue were observed in all the initial bindings (K156 in MD2 and MD3 made first contact as well).

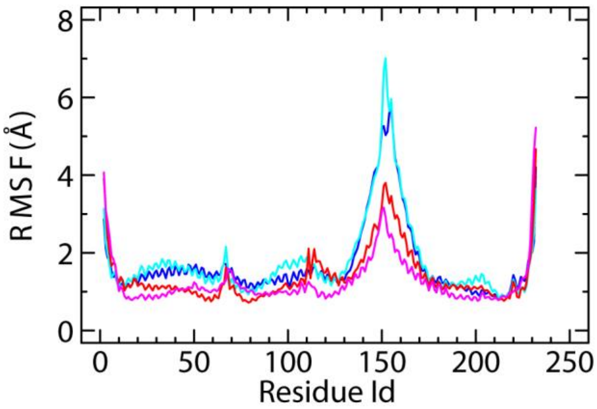

Figure S1. Root-mean-square-fluctuation (RMSF) of C_α atoms.

RMSF obtained from the last 50-ns MD simulation of I-BAR domain dimer in solution (blue and cyan) and MD1 (red and magenta). The peaks around residue 150-155 indicate large fluctuation at the tips of I-BAR domain.

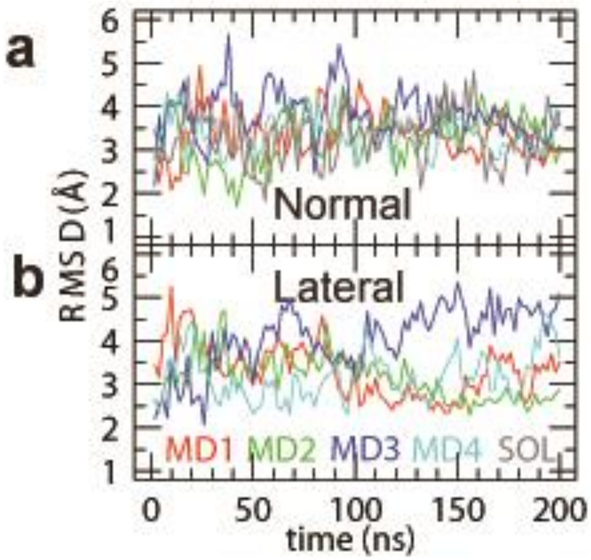

Figure S2. Root mean square deviation (RMSD) from the crystal structure.

RMSD of heavy atoms from the crystal structure along the membrane bilayer (*a*) normal and (*b*) lateral direction.

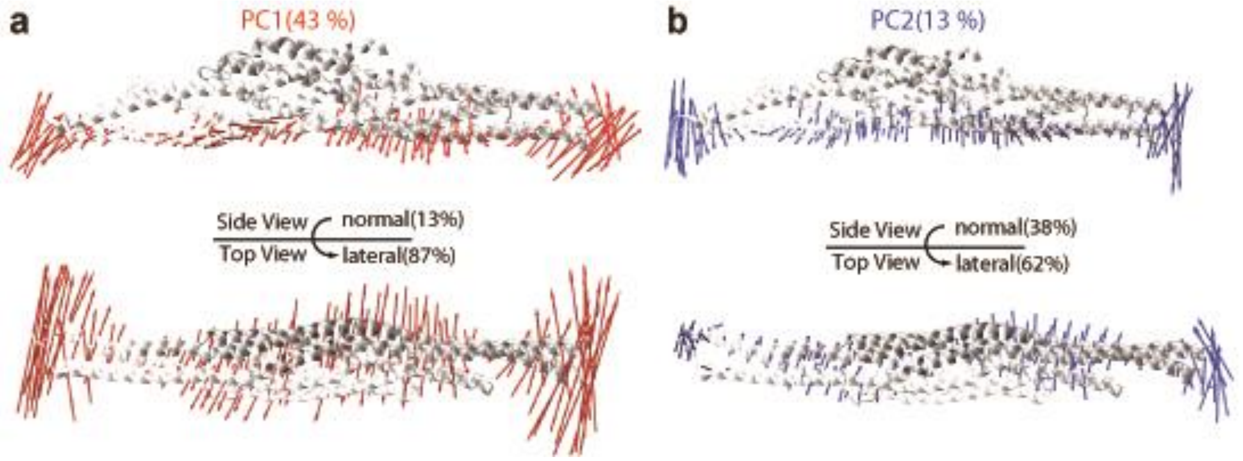

Figure S3. Principal components obtained from MD simulation in solution.

*(a)*The first and (*b*) second principal components obtained from the MD simulation in solution. The principal component analysis was conducted for the last 100 ns MD, adopting the heavy atoms of the lipid binding residues. The molecular graphics was created by VMD[5].

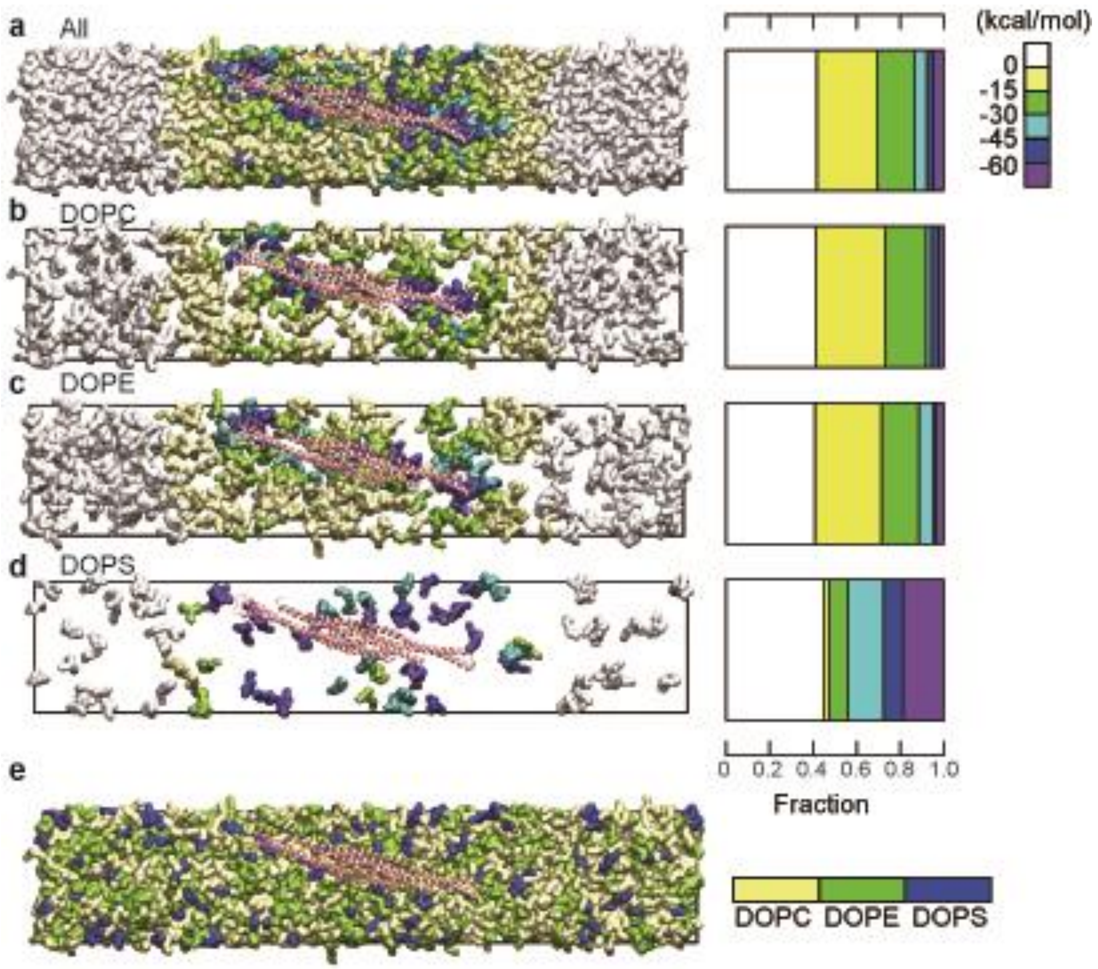

Figure S4. Interaction energy of I-BAR with the lipid headgroups.

Interaction energy of I-BAR with each *(a)* all, *(b)* DOPC, *(c)* DOPE, and *(d)* DOPS lipid headgroup. The interaction energy was calculated for the MD1 trajectory of during 107~137 ns. Color bars show fraction of lipid head groups within each range of the interaction energy. *(e)* Snapshots at 123 ns from MD1. Yellow, green, and blue represent the headgroups of DOPC, DOPE, and DOPS, respectively. The molecular graphics was created by VMD[5].

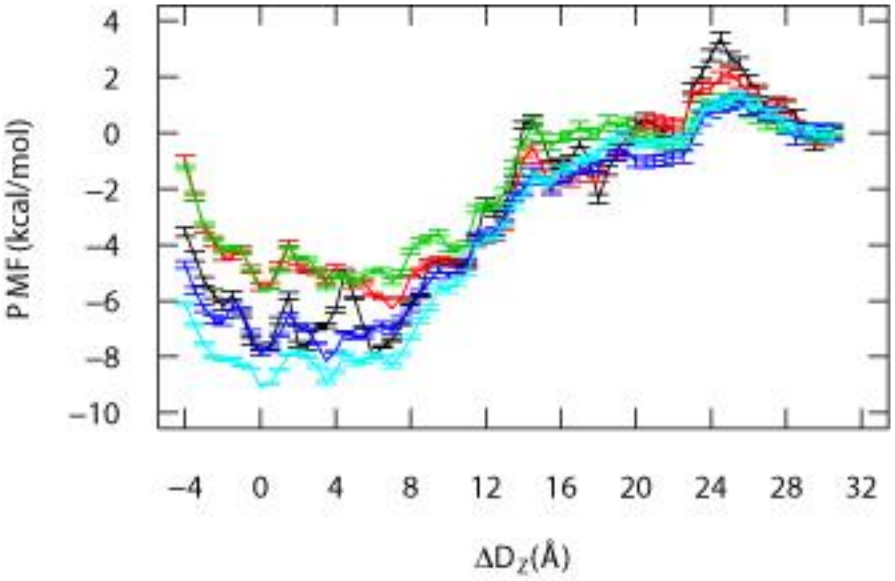

Figure S5. Convergence of umbrella sampling simulations.

Binding free energy ΔG_B obtained from 2 (black), 4 (red), 6 (green), 8 (blue) and 10 (cyan) ns MD simulations.

Movie S1. The I-BAR binding to the membrane and deformation of the membrane in MD1.

Movie S2. The I-BAR binding to the membrane and deformation of the membrane in MD2.

Movie S3. The I-BAR binding to the membrane and deformation of the membrane in MD3.

Movie S4. The I-BAR binding to the membrane and deformation of the membrane in MD4.

In each movie, top (upper) and side (lower) view are shown. Lipid headgroups are represented by phosphorus atoms using sphere with van der Waals radius. For top views, only upper leaflet of lipid bilayer is shown, and periodic images lipids are shown. Yellow color represents lipids in contact with I-BAR. The movies were created by VMD[5].

References

1 Suetsugu, S. *et al.* The RAC binding domain/IRSp53-MIM homology domain of IRSp53 induces RAC-dependent membrane deformation. *J. Biol. Chem.* **281**, 35347-35358 (2006).

2 Karplus, M. & Kushick, J. N. Method for Estimating the Configurational Entropy of Macromolecules. *Macromolecules* **14**, 325-332 (1981).

3 Schlitter, J. Estimation of Absolute and Relative Entropies of Macromolecules Using the Covariance-Matrix. *Chem. Phys. Lett.* **215**, 617-621 (1993).

4 Takemura, K. *et al.* Free-energy analysis of lysozyme-triNAG binding modes with all-atom molecular dynamics simulation combined with the solution theory in the energy representation. *Chem. Phys. Lett.* **559**, 94-98 (2013).

5 Humphrey, W., Dalke, A. & Schulten, K. VMD: visual molecular dynamics. *J. Mol. Graph.* **14**, 33-38, 27-38 (1996).
